# Cost-effectiveness and Pricing of Antibacterial Drugs

**DOI:** 10.1111/cbdd.12417

**Published:** 2014-12-17

**Authors:** Talitha I Verhoef, Stephen Morris

**Affiliations:** Department of Applied Health Research, University College LondonGower Street, London, WC1E 6BT, UK

**Keywords:** antibacterial drugs, antibiotics, cost-effectiveness, health economics, value-based pricing

## Abstract

Growing resistance to antibacterial agents has increased the need for the development of new drugs to treat bacterial infections. Given increasing pressure on limited health budgets, it is important to study the cost-effectiveness of these drugs, as well as their safety and efficacy, to find out whether or not they provide value for money and should be reimbursed. In this article, we systematically reviewed 38 cost-effectiveness analyses of new antibacterial agents. Most studies showed the new antibacterial drugs were cost-effective compared to older generation drugs. Drug pricing is a complicated process, involving different stakeholders, and has a large influence on cost-effectiveness. Value-based pricing is a method to determine the price of a drug at which it can be cost-effective. It is currently unclear what the influence of value-based pricing will be on the prices of new antibacterial agents, but an important factor will be the definition of ‘value’, which as well as the impact of the drug on patient health might also include other factors such as wider social impact and the health impact of disease.

Since the discovery of the first antibacterial agents in the 1930s, many new antibacterial drugs have been developed. This has had a large influence on health because of the decreased mortality and morbidity of bacterial infections 1,2. Between the 1970s and the 1990s, few new antibacterial drug classes were launched 3,4. Pharmaceutical companies were reluctant to invest in the development of antibacterial agents; the revenues of the new drugs were expected to be low, because of the short use of antibacterial drugs and the high competition with many cheap generic drugs 3,5. The lack of new antibacterial agents and the growing resistance to the available drugs has limited the treatment options for infection in recent years. However, since 2000, a series of new classes of antibacterial drugs such as oxazolidinones and lipopeptides have been launched 2–4. Increasingly, cost-effectiveness is an important factor affecting the reimbursement of new drugs,^a^ and it is important to investigate whether these drugs provide value for money. In this article, we will describe and explain some basic concepts of cost-effectiveness and review the available evidence on the cost-effectiveness of new antibacterial agents. Also, we will provide a brief historical overview of drug pricing (in the UK) and describe the role of value-based pricing in determining the price of new antibacterial agents.

## What is Cost-effectiveness Analysis?

**Figure 1:**
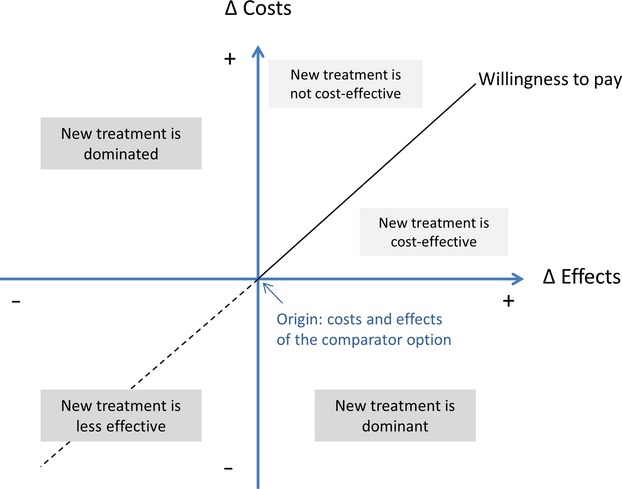
Cost-effectiveness plane depicting the incremental costs and incremental effects of the new treatment versus the comparator.

In a cost-effectiveness analysis (CEA), the total costs and effects of two or more treatment options are compared. When calculating costs, not only should the costs of the drugs be considered, including administration costs and costs of treating adverse drug reactions, but also other costs related to the treatment or disease, such as hospitalization costs. To study cost-effectiveness, both incremental (extra) costs and incremental effects are calculated for the new treatment option compared to the comparator option, which might be current best practice and which might be ‘do nothing’. Figure1 depicts the possible results of a CEA. Compared with the comparator option, the new treatment may be more or less costly and more or less effective. When the new treatment leads to increased effects while decreasing costs (bottom right quadrant), it is the dominant treatment option, which means that it is more attractive than the comparator on economic grounds. When the new treatment leads to decreased effect while increasing costs (top left quadrant), it is dominated by the comparator, which means that the comparator is a more attractive option. When both costs and effects are increased (top right quadrant), the attractiveness of the new treatment depends on how much payers are prepared to pay for the extra effect. When the incremental costs per extra unit of effect are lower than the willingness-to-pay threshold (top right quadrant below the 45 degree line), the new treatment is cost-effective. Conversely, when the incremental costs per extra unit of effect are higher than the threshold (top right quadrant above the 45 degree line), the new treatment is not cost-effective. When costs and effects are higher with a new drug compared with the comparator, the incremental cost-effectiveness ratio (ICER) can be calculated as shown in the equation below.

Calculation of the ICER

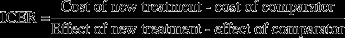


Effectiveness can be measured in many ways usually related to the treatment goal of the drug (e.g. cure rate). When effectiveness is measured in terms of cure rates, cost-effectiveness measured using the ICER is expressed as the incremental cost per extra case cured. This outcome measure is, however, very disease-specific, and therefore, it is difficult to compare with treatments in other diseases. A recommended measure is quality-adjusted life-years (QALYs), which account for both quality and quantity of life.^b^ There are various ways of measuring the quality of life, including direct valuation methods such as standard gamble, time trade-off and visual analogue scale 6 or questionnaires such as the EQ-5D 7. When effects are measured by QALYs, the ICER is the incremental cost per QALY gained, and as this is a generic health outcome measure, it can facilitate comparisons between treatments for different diseases. For this reason, some regulatory authorities or health insurance companies require evidence of a favourable incremental cost per QALY gained before they approve the use of a new drug^a^,^b^. Another generic measure is life-years gained which is based on life expectancy only and does not take into account quality of life. This measure is easier to calculate, because it does not require information on health-related quality of life impacts, but it is not as comprehensive as QALYs.

Whether or not a new treatment is cost-effective depends on the decision-makers' willingness to pay for a QALY, which is different across different settings. In the USA, a threshold of US$50 000–100 000 per QALY gained is commonly reported 8; in the UK, a range of £20 000–30 000 is used^b^. Two important issues when considering cost-effectiveness of a new intervention are perspective and time horizon^b^. The perspective is the viewpoint from which the analysis is performed (e.g. patient, hospital, healthcare system, society), and this influences the type of costs that should be collected (healthcare costs, patient-borne costs, costs borne by the rest of society, etc.). The time horizon should reflect the period over which the main differences between two interventions are expected. In many cases, this is a lifetime horizon. When the time horizon is more than 1 year, discounting of costs and effects is usually recommended, which has the effect of giving less weight to costs and benefits that occur in the future. The recommended discount rates for costs and effects vary by country, for example, in the UK the discount rates for both costs and effects should be 3.5% and in the Netherlands the discount rate for costs should be 4% and the discount rate for effects only 1.5%^a^.

Every CEA has some level of uncertainty around the results. This can be because of uncertainty around the effectiveness estimate or around the costs. There may also be variability in costs and effects between patients, for example, because of differences in severity of the disease. This uncertainty should be assessed in a sensitivity analysis. In a one-way sensitivity analysis, the value of one parameter is varied over a plausible range, to show the effect of this parameter on the cost-effectiveness results. This can be performed for several or all parameters, varying only one at a time. It is the simplest form of sensitivity analysis and is useful to identify the parameters with the largest influence on the results. It is, however, not regarded as sufficient because it does not take into account any combined effect of parameters. In a two-way (or multiway) sensitivity analysis, two (or several) parameters are varied at the same time to assess their joint influence on the results.

Probabilistic sensitivity analysis is a technique that can be used to investigate joint parameter uncertainty. It can be used to generate cost-effectiveness acceptability curves, showing the chance that a drug would be cost-effective at a range of cost-effectiveness threshold values. This can be performed by carrying out a large number of simulations, drawing random samples from probability distributions for the ranges of key parameters. The number of simulations below the cost-effectiveness threshold represents the chance that the drug would be cost-effective given that threshold.

Other forms of sensitivity analysis include threshold analysis (identifying a threshold value for a parameter at which the new treatment would just be cost-effective) and scenario analysis (for example, showing a best-case scenario and a worst-case scenario).

## Cost-effectiveness of New Antibacterial Agents

Since 2000, 22 new antibacterial agents have become available on the market (Box 1) 2. In this section, we will review the available evidence on the cost-effectiveness of these new drugs, in comparison to the older antibacterial agents. Using the NHS Economic Evaluation Database (NHS EED),^c^ we performed an initial search for CEAs of all 22 new drugs. Using the drugs in Box 1 as search terms, we identified 41 records. Of these, 12 were excluded, three studies were published in a non-English language 9–11, one did not concern any of the new drugs 12 and eight did not link costs and effects explicitly 13–20. Hence, an initial 29 studies were included for review (21–48,58, 58). We then undertook a more extensive search using Embase and PubMed using the same search terms including cost-effectiveness terms and found nine additional papers 49–57. Only studies reporting both costs and effects of the new drug compared to the comparator were included. Conference abstracts and non-English papers were excluded. From the papers included for review, we collected the following information: year of study, comparator, disease, outcome measure, whether sensitivity analysis was performed to assess uncertainty and which type of sensitivity analysis and final result (cost-effective or not). We used the PRISMA guidelines for systematic searching.

**Box 1: New antibiotics launched since 2000 2**

**Table:** 

2000 Linezolid	2004 Gemifloxacin	2009 Tebipenem pivoxil
2001 Telithromycin	2005 Doripenem	2009 Telavancin
2002 Biapenem	2005 Tigecycline	2009 Antofloxacin
2002 Ertapenem	2007 Retapamulin	2009 Besifloxacine
2002 Prulifloxacin	2007 Garenoxacin	2010 Ceftaroline fosamil
2002 Pazufloxacin	2008 Ceftobiprole medocaril	2011 Fidaxomicin
2002 Balofloxacin	2008 Sitafloxacin	2012 Bedaquiline
2003 Daptomycin

Several CEAs have been published on linezolid, which was launched in 2000. Other drugs that have been evaluated are daptomycin, telithromycin, ertapenem, gemifloxacin, doripenem, telavancin and fidaxomicin (see Table1).

**Table 1:** Reviewed cost-effectiveness studies on antibacterial agents

Year	Comparator	Disease	Outcome measure	Uncertainty analysis	Cost-effective?	Ref
Linezolid						
2001	Flucloxacillin or vancomycin	Cellulitis	Cost per extra cure/success	1-way	Yes	55
2003	Oxacillin or vancomycin	Cellulitis	Cost per extra cure/success	1-way	Yes	47
2004	Vancomycin	Pneumonia	Cost per QALY gained	1-way, two-way, PSA	Yes	43
2005	Vancomycin	Pneumonia	Cost per QALY gained	Scenario	Yes	49
2005	Teicoplanin	Gram-positive bacteraemia	Cost per extra cure/success	1-way, PSA	Yes	30
2005	Vancomycin	MRSA pneumonia	Cost per extra cure/success	No	Unsure	36
2006	Vancomycin	MRSA pneumonia	Cost per life saved	1-way, PSA	Yes	37
2007	Teicoplanin	Gram-positive infections	Cost per extra cure/success	1-way	Yes	29
2007	Vancomycin	MRSA surgical site infections	Cost per extra cure/success	1-way, threshold	Yes	40
2008	OPAT	Skin infections	Cost per extra cure/success	No	Yes	44
2009	Vancomycin	MRSA skin infections	Cost per extra cure/success	1-way, PSA	Unsure	23
2009	Vancomycin	MRSA pneumonia	Cost per life-year gained	1-way, 2-way	Yes	27
2009	Vancomycin	MRSA skin infections	Cost per extra cure/success	Scenario, 1-way, 2-way	Yes	28
2009	Vancomycin	Skin infections	Cost per extra cure/success	1-way, PSA	Yes	42
2014	Vancomycin	MRSA pneumonia	Cost per extra cure/success	Scenario	Yes	54
Daptomycin						
2007	Vancomycin	Skin infections	Cost per extra cure/success	No	Yes	26
2009	Vancomycin–gentamicin	MRSA bacteremia/endocarditis	Cost per extra cure/success	Scenario	Unsure	22
Linezolid & Daptomycin						
2011	Vancomycin	MRSA skin infections	Cost per extra cure/success	Scenario, 1-way, PSA	Yes	24
2012	TMP/SMX	MRSA infections	Cost savings	No	No	25
2014	Vancomycin or *β*-lactam	Enterococcal bacteremia	Cost per QALY gained	1-way, PSA	Yes	52
Telithromycin						
2004	Clarithromycin	Pneumonia	Cost savings	Subgroup analysis	Yes	38
2004	Clarithromycin	Pneumonia	Cost savings	No	Unsure	39
2004	Clarithromycin	Pneumonia	Cost savings	No	Unsure	46
2008	Several comparators^a^	Pneumonia	Cost per extra cure/success	1-way	No	41
Ertapenem						
2008	Cefotetan	Prophylaxis before surgery	Cost per failure avoided	No	Yes	57
2009	Piperacillin/tazobactam	Intra-abdominal infections	Cost per QALY gained	Scenario, 1-way, PSA	Yes	32
2009	Piperacillin/tazobactam	Diabetic foot infections	Cost per QALY gained	Scenario, PSA	Yes	33
2014	Ceftriaxone	Pneumonia	Cost per extra cure/success	1-way, PSA	Yes	50
Gemifloxacin						
2002	Clarithromycin	Exacerbations of chronic bronchitis	Cost per extra cure/success	Scenario, PSA	Yes	31
2008	Ceftriaxone/cefuroxime	Pneumonia	Cost per extra cure/success	No	Unsure	21
Doripenem						
2010	Imipenem	Pneumonia	Cost savings	1-way	Yes	34
2010	Imipenem	Pneumonia	Cost savings	1-way, PSA	Yes	53
2010	Imipenem-cilastatin	Pneumonia	Cost per QALY gained	Scenario, 1-way, 2-way, PSA	Yes	48
Telavancin						
2008	Vancomycin	Skin infections	Cost per extra cure/success	1-way, PSA	Yes	35
Fidaxomicin						
2013	Vancomycin or metronidazole	Clostridium difficile infection	Cost per QALY gained	1-way, two-way, PSA	No	58
2013	Vancomycin	Clostridium difficile infection	Cost per QALY gained	1-way, PSA	Yes	45
2014	Vancomycin, metronidazole or FMT	Recurrent clostridium difficile infection	Cost per QALY gained	1-way, two-way, PSA	No	51
2014	Vancomycin	Clostridium difficile infection	Cost per recurrence avoided	1-way	Unsure	56

OPAT, Outpatient parenteral antimicrobial therapy; TMP/SMX, Trimethoprim/sulfamethoxazole; PSA, probabilistic sensitivity analysis; FMT, faecal microbiota transplant; QALY, quality-adjusted life-years.

a Moxifloxacin, amoxicillin or clarithromycin.

Cost-effectiveness was always assessed for one specific indication, such as pneumonia or skin infections caused by methicillin-resistant staphylococcus aureus (MRSA) or by other bacteria. Most of the published studies assessed the incremental cost per extra case cured with the new antibacterial drug. When both the costs and the cure rate of the new drug were higher than the comparator (top right quadrant in Figure1) 21–23,36,56, it was difficult to assess whether the new drug was cost-effective or not, because there is no willingness-to-pay threshold for cost per extra case cured. In many studies, the new drugs were more effective, and total costs were lower, which means that the new treatment was the dominant treatment (bottom right quadrant in Figure1). In this case, we can conclude that the new drug was cost-effective. In some studies, the authors found that the effectiveness of the new drug and the comparator were equal, and therefore, they performed a cost minimization study, only looking at the cost of the drug versus the comparator.

Nine studies assessed the incremental cost per QALY gained for linezolid, daptomycin, ertapenem, doripenem and fidaxomicin 32,33,43,45,48,49,51,52,58. In seven of these studies, the ICER was below the willingness-to-pay threshold. In one of these studies, the incremental cost per QALY gained of fidaxomicin compared to vancomycin was US$67 576 45. The authors applied a willingness-to-pay threshold of US$100 000, so this drug was considered cost-effective for the treatment of *Clostridium difficile* infections.

Most studies showed that the new drugs were cost-effective, except in four cases. Both linezolid and daptomycin were dominated by trimethoprim/sulfamethoxazole for the treatment of MRSA infections 25, and telithromycin was dominated by moxifloxacin for the treatment of pneumonia 41. In two studies, on incremental cost per QALY gained, fidaxomicin was dominated by one or more of the comparators 51,58. All studies correctly considered not only the costs of the drug itself, but also other costs, such as hospitalization costs, which can affect the ICER.

Because uncertainty is present in every cost-effectiveness study, a good economic evaluation should perform a sensitivity analysis to examine the robustness of the model and assumptions. In some studies, no sensitivity analysis was performed, but most studies included at least some form of sensitivity analysis. Almost half of the studies included a probabilistic sensitivity analysis.

Some antibacterial agents can be used for various indications. The cost-effectiveness, however, has mostly been studied for one indication at a time. The cost-effectiveness of the drug can vary between different indications, because of different bacteria causing the infection or because of differences in location of the bacteria. It is therefore necessary to assess the cost-effectiveness of a new drug for a specific indication, even if the drug can be used for more than one indication. The cost-effectiveness of a specific drug might also change over time, when for example the resistance to the old drugs increases or when resistance to the new drug develops. The growing resistance to antibacterial agents highlights the urgent need for the development of new drugs to fight infections.

## Value-based Pricing

Pricing of antibacterial drugs is an important topic to consider. For pharmaceutical companies, it is important to make profit on the sales of these drugs, because of the large investments needed to develop a new drug. But due to the limited budget, and the rising proportion of health spending that is accounted for by pharmaceuticals, good value for money is an important factor before reimbursement of a new drug. Drug prices are often an important driver of cost-effectiveness. Additionally, infections occur more frequently in developing countries with low insurance coverage where many patients cannot afford expensive antibacterial drugs. Pricing of the drugs is therefore a complicated process, involving different stakeholders.

Box 2 shows a historical overview of pharmaceutical pricing in the UK 59. In this section, we provide a brief summary of this, focusing in particular on value-based pricing. The timelines and examples are UK specific, but could easily be applied elsewhere. Many countries use cost-effectiveness analysis in a similar way to inform reimbursement decisions^a^, although precise guidelines for undertaking such evaluations may vary by country.

**Box 2: Historical overview of pharmaceutical pricing in the UK 59**

**Table:** 

1957	Voluntary Pricing Regulation Scheme established
1978	Voluntary Pricing Regulation Scheme was renamed to Prescription Pricing Regulation Scheme (PPRS)
1999	NICE established
2002	NICE advices NHS not to provide new multiple sclerosis drugs. The government establishes a risk-sharing scheme giving patients access to these drugs
2007	Office of Fair Trading recommends value-based pricing for all branded drugs
2008	Government negotiates a scope for price reductions on particular drugs through patient access schemes.
2009	NICE's end of life criteria raise the willingness-to-pay threshold for these drugs
2010	Government commitment to value-based pricing in next PPRS
2011	Government response to consultation on value-based pricing indicates that it will apply only to new drugs and give greater role to NICE
2013	Government response to House of Commons Health Committee report confirms NICE to take responsibility for value-based pricing
Late 2014	Value-based pricing for new drugs appraised by NICE (expected)

In the UK, since 1999, the National Institute for Health and Care Excellence (NICE) appraises the clinical effectiveness and cost-effectiveness of new drugs. Although this body has no direct influence on the price, they do give advice about whether or not drugs (and other healthcare and public health interventions) should be provided by the National Health Service (NHS). NICE accounts for several factors in its decision-making process, but when the incremental costs per QALY gained are higher than £30 000, NICE normally advises not to include this treatment in the NHS setting^b^. As noted, a key driver of cost-effectiveness is drug price. The Prescription Pricing Regulation Scheme (PPRS), established in 1957 as the Voluntary Pricing Regulation Scheme, usually negotiates with pharmaceutical companies to have drugs with a reasonable price which are still profitable for the pharmaceutical industry. When NICE would consider a new drug not cost-effective at that price, pharmaceutical companies may be willing to negotiate a lower price in order to be able to sell the drug in the NHS setting. In 2002, the government established a risk-sharing scheme for new multiple sclerosis drugs. These drugs could be sold at a high price, but if the outcomes of a cohort study were lower than expected, the price was to be reduced. This way, the risk of the drug not being cost-effective was shared by the NHS and the drug company. Since 2009, it is possible for pharmaceutical companies to negotiate price reductions when the drug is considered not cost-effective by NICE and therefore not available on the NHS. A discount is provided, so that the costs of the new treatment are below the threshold set by NICE and the treatment can be available on the NHS (patient access schemes) 59. Also, NICE increased the willingness-to-pay threshold for drugs extending the life of patients with a short life expectancy (end of life criteria).

**Figure 2:**
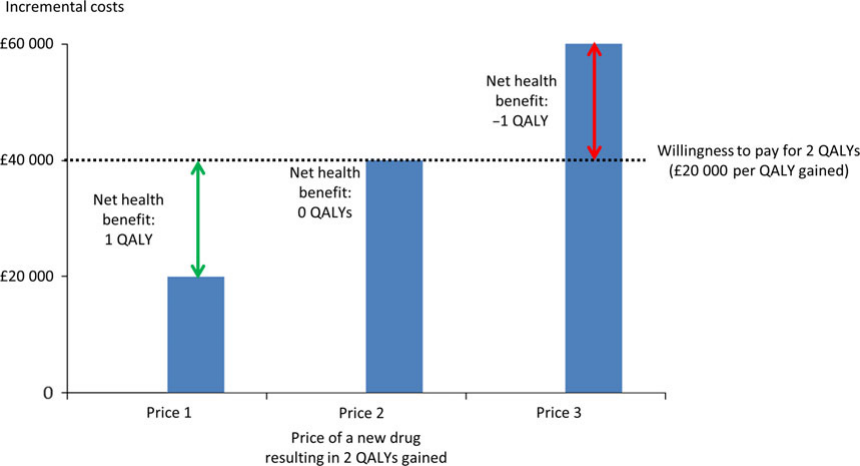
Net health benefit of a hypothetical new drug at three different prices 60.

Instead of studying the cost-effectiveness of a drug with a certain price, NICE could also calculate the maximum price of the drug at which it would still be cost-effective. When the price of the drug would be no higher than this threshold value, it would represent ‘value for money’. Given the fixed budget of the NHS, money spent on a new drug cannot be spent elsewhere. With a willingness-to-pay threshold of £20 000 per QALY gained, we assume that every £20 000 used for a new treatment displaces one QALY elsewhere in the NHS 60. The QALYs expected to be displaced by a new treatment can be calculated by dividing the total expected costs by the willingness-to-pay value. This forms the basis of value-based pricing. Figure2 depicts the costs of a hypothetical new drug that increases health by 2 QALYs compared with the comparator at three different prices. At price 1, the total extra cost of this treatment to the NHS is £20 000, which leads to an ICER of £10 000 per QALY gained. So, at price 1, the drug is expected to improve health by two QALYs and displace 1 QALY (total costs of £20 000 divided by the willingness to pay of £20 000) elsewhere in the NHS. The net health benefit, defined as the difference between the total expected QALYs and the QALYs expected to be displaced elsewhere, would in this case be one QALY (2 expected QALYs minus 1 QALY displaced elsewhere). At price 2, the total extra costs of this treatment are £40 000, which leads to an ICER of £20 000 per QALY gained. Now the drug is still expected to improve health by 2 QALYs, but displaces 2 QALYs (£40 000) elsewhere. There is no net health benefit to the NHS. At price 3, the total extra costs of the treatment are £60 000, which leads to an ICER of £30 000 per QALY. In this case, 3 QALYs are displaced (£60 000), and the net health benefits to the NHS are minus 1 QALY (2 expected QALYs minus 3 QALYs forgone elsewhere). The aim of value-based pricing is to set the price at a level at which the net health benefit is positive or zero. A negative health benefit means that the drug does not provide value for money. Given that the definition of net health benefit is the difference between the total expected QALYs and the QALYs expected to be displaced elsewhere (total expected costs divided by the willingness-to-pay value), one could also say that the aim of value-based pricing is to set the price of a new drug to a value at which the ICER of that new drug would be at or below the willingness-to-pay threshold. In other words, the price can be varied up to the point at which the incremental cost per QALY is equal to the threshold.

Value-based pricing was first recommended by the Office of Fair Trading.^d^ A key issue with value-based pricing is how ‘value’ is measured. One option would be to express value as therapeutic value, measured by QALYs. When a new drug is very effective (it produces a large increase in QALYs), the drug can be more expensive than when it is moderately effective (small increase in QALYs). However, it has been suggested that not only QALYs should be taken into account when assessing value, and many other factors could also be included (see Box 3) 61,62. Based on recent consultation documents produced by NICE,^e^ two additional components of value that are being given careful consideration are the burden of disease and the wider social impact – the impact of disease on people's ability to be part of society. Whichever values are included in value-based pricing, an important consideration is how these ought to be weighted to account for the fact that different drugs may have different effects on each factor.

**Box 3: Possible factors to take into account when assessing value 61,62**

**Table:** 

Patient or disease-related factors	Healthcare process-related factors	Factors outside patient + NHS
Severity of disease	Treatment time + location	Ability to resume working
Near the end of life	Waiting times	Increased productivity
Size of population	Less unpleasant treatment	Benefit to carers
No other treatment options	Degree of risk of the treatment	Cost savings to other services
Socially disadvantaged patients		Cost savings to patients/carers
Children		Quality of evidence
Reduction in fear (e.g. of death)		Innovation
Unmet need

There is little evidence from other countries about the prospects of value-based pricing, because no country currently performs value-based pricing as proposed in the UK. However, in some countries, the insurance coverage of a new drug is based on the cost-effectiveness of the drug. In Sweden, insurance coverage depends on approval from their health technology assessment body (TLV). For this decision, QALYs as well as production loss are taken into account.^f^

## Future Prospects

It is unclear yet what the influence of value-based pricing will be on the prices of new antibacterial agents. If we look at the different components NICE is planning to consider in their evaluation, three factors will be of importance; health gain (QALYs), burden of disease and wider social impact.

### Health gain

Many of the studies reviewed in this article showed that the new antibacterial agents were more effective than the comparator, mostly because of a higher cure rate. Because these drugs can be life-saving, a large ‘value’ can be expected for which a high price can be paid. However, there is currently little evidence about the incremental costs per QALY gained. More evidence is needed using the effect of new antibacterial agents on QALYs to set a value-based price for these drugs.

### Burden of disease

Because bacterial infections occur frequently, the total burden of these infections is large. However, as we have seen in this review, each indication (type of infection) needs to be considered separately. For some indications, the burden of disease might be lower than others. In general, the burden of one specific bacterial infection is probably low, also because of the short duration of the illness. Chronic conditions have a more significant effect on burden of disease than (treatable) acute conditions.

### Wider social impact

National Institute for Health and Care Excellence defines the wider social impact as the loss in capacity of a person with the disease to engage with society (e.g. working or taking care of someone), compared with their capacity without the disease. If infections occur among working people, the impact on productivity might be high. However, when the duration of the infection is usually short, the impact will not be as high as for example with chronic diseases.

## Conclusions

Since 2000, several new antibacterial agents have been developed to treat (resistant) infections such as pneumonia and skin infections. To keep healthcare spending within reasonable limits, it is important that new drugs are only prescribed when these drugs provide value for money. Most new antibacterial agents are cost-effective alternatives to the old drugs, either because these are more effective and decrease healthcare costs or the increased costs are below the willingness-to-pay threshold. Value-based pricing is a method that could be used to determine a price for new antibacterial agents at which these drugs provide value for money.

## Conflict of interest

None declared.

## Notes

a http://www.ispor.org/peguidelines/index.asp

b NICE National Institute of Health and Care Excellence. Guide to the methods of technology appraisal 2013 (Available from: http://www.nice.org.uk/media/D45/1E/GuideToMethodsTechnologyAppraisal2013.pdf).

c NHS Economic Evaluation Database (NHS EED). Available at http://www.crd.york.ac.uk/crdweb/.

d (2007) Office of Fair Trading. The pharmaceutical price regulation scheme: an OFT market study (Available at: http://www.oft.gov.uk/shared_oft/reports/comp_policy/oft885.pdf).

e NICE National Institute of Health and Care Excellence, Centre for health technology evaluation. Value Based Assessment of Health Technologies. Consultation paper. (Available from: http://www.nice.org.uk/media/FE2/2B/VBA_TA_Methods_Guide_for_CONSULTATION_upload.pdf).

f Persson U (2012) Value based pricing in Sweden: Lessons for design? OHE briefing No 12. (available at: http://www.ohe.org/publications/article/value-based-pricing-in-sweden-122.cfm).

## References

[1] Zaffiri L, Gardner J, Toledo-Pereyra LH (2012). History of antibiotics. From salvarsan to cephalosporins. J Invest Surg.

[2] Butler MS, Blaskovich MA, Cooper MA (2013). Antibiotics in the clinical pipeline in 2013. J Antibiot (Tokyo).

[3] Norrby SR, Nord CE, Finch R (2005). Lack of development of new antimicrobial drugs: a potential serious threat to public health. Lancet Infect Dis.

[4] Outterson K, Samora JB, Keller-Cuda K (2007). Will longer antimicrobial patents improve global public health?. Lancet Infect Dis.

[5] Projan SJ (2003). Why is big Pharma getting out of antibacterial drug discovery?. Curr Opin Microbiol.

[6] Torrance GW (1986). Measurement of health state utilities for economic appraisal. J Health Econ.

[7] EuroQol Group (1990). EuroQol – a new facility for the measurement of health-related quality of life. Health Policy.

[8] Shiroiwa T, Sung YK, Fukuda T, Lang HC, Bae SC, Tsutani K (2010). International survey on willingness-to-pay (WTP) for one additional QALY gained: what is the threshold of cost effectiveness?. Health Econ.

[9] Redondo E, Nocea G (2003). Cost-effectiveness analysis of ertapenem (Invanz) in intra-abdominal infections compared to piperacillin/tazobactam. Revista Espanola de Economia de la Salud.

[10] Rubio-Terres C, Cots JM, Dominguez-Gil A, Herreras A, Sanchez GF, Chang J, Trilla A (2003). Pharmacoeconomic analysis of community-acquired pneumonia treatment with telithromycin or clarithromycin. Rev Esp Quimioter.

[11] Rubio-Terres C, Cots JM, Dominguez-Gil A, Herreras A, Sanchez Gascon F, Chang J, Trilla A (2004). Pharmacoeconomic analysis of patients with acute exacerbation of chronic bronchitis treated with telithromycin or cefuroxime-axetil. Rev Clin Esp.

[12] Phillips S, MacDougall C, Holdford DA (2007). Analysis of empiric antimicrobial strategies for cellulitis in the era of methicillin-resistant *Staphylococcus aureus*. Ann Pharmacother.

[13] Brown KR, Williams SF, Apuzzio JJ (2012). Ertapenem compared to combination drug therapy for the treatment of postpartum endometritis after cesarean delivery. J Matern Fetal Neonatal Med.

[14] Gonzalez-Ruiz A, Richardson J (2008). Are glycopeptides still appropriate and convenient for empiric use?. J Chemother.

[15] Hermsen ED, Shull SS, Mitropoulos IF, Puumala SE, Rupp ME (2009). Prospective evaluation of clinical and economic outcomes associated with treatment of serious infections due to gram-positive cocci. Infect Dis Clin Pract.

[16] Kullar R, Davis SL, Kaye KS, Levine DP, Pogue JM, Rybak MJ (2013). Implementation of an antimicrobial stewardship pathway with daptomycin for optimal treatment of methicillin-resistant *Staphylococcus aureus* bacteremia. Pharmacotherapy.

[17] McKinnon PS, Carter CT, Girase PG, Liu LZ, Carmeli Y (2007). The economic effect of oral linezolid versus intravenous vancomycin in the outpatient setting: the payer perspective. Manag Care Interface.

[18] Merchant S, Gast C, Nathwani D, Lee M, Quintana A, Ketter N, Friedland I, Ingham M (2008). Hospital resource utilization with doripenem versus imipenem in the treatment of ventilator-associated pneumonia. Clin Ther.

[19] Sclar DA, Robison LM, Oganov AM, Schmidt JM, Bowen KA, Castillo LV (2012). Fidaxomicin for *Clostridium difficile*-associated diarrhoea: epidemiological method for estimation of warranted price. Clin Drug Investig.

[20] Wright BM, Eiland EH (2011). Retrospective analysis of clinical and cost outcomes associated with methicillin-resistant *Staphylococcus aureus* complicated skin and skin structure infections treated with daptomycin, vancomycin, or linezolid. J Pathog.

[21] Bhavnani SM, Ambrose PG (2008). Cost-effectiveness of oral gemifloxacin versus intravenous ceftriaxone followed by oral cefuroxime with/without a macrolide for the treatment of hospitalized patients with community-acquired pneumonia. Diagn Microbiol Infect Dis.

[22] Bhavnani SM, Prakhya A, Hammel JP, Ambrose PG (2009). Cost-Effectiveness of daptomycin versus vancomycin and gentamicin for patients with methicillin-resistant *Staphylococcus aureus* bacteremia and/or endocarditis. Clin Infect Dis.

[23] Bounthavong M, Hsu DI, Okamoto MP (2009). Cost-effectiveness analysis of linezolid vs. vancomycin in treating methicillin-resistant *Staphylococcus aureus* complicated skin and soft tissue infections using a decision analytic model. Int J Clin Pract.

[24] Bounthavong M, Zargarzadeh A, Hsu DI, Vanness DJ (2011). Cost-effectiveness analysis of linezolid, daptomycin, and vancomycin in methicillin-resistant *Staphylococcus aureus*: complicated skin and skin structure infection using Bayesian methods for evidence synthesis. Value Health.

[25] Campbell ML, Marchaim D, Pogue JM, Sunkara B, Bheemreddy S, Bathina P, Pulluru H (2012). Treatment of methicillin-resistant *Staphylococcus aureus* infections with a minimal inhibitory concentration of 2 mug/mL to vancomycin: old (trimethoprim/sulfamethoxazole) versus new (daptomycin or linezolid) agents. Ann Pharmacother.

[26] Davis SL, McKinnon PS, Hall LM, Delgado G, Rose W, Wilson RF, Rybak MJ (2007). Daptomycin versus vancomycin for complicated skin and skin structure infections: clinical and economic outcomes. Pharmacotherapy.

[27] De Cock E, Krueger WA, Sorensen S, Baker T, Hardewig J, Duttagupta S, Muller E, Piecyk A, Reisinger E, Resch A (2009). Cost-effectiveness of linezolid vs vancomycin in suspected methicillin-resistant *Staphylococcus aureus* nosocomial pneumonia in Germany. Infection.

[28] De Cock E, Sorensen S, Levrat F, Besnier JM, Dupon M, Guery B, Duttagupta S (2009). Cost-effectiveness of linezolid versus vancomycin for hospitalized patients with complicated skin and soft-tissue infections in France. Med Mal Infect.

[29] Grau S, Aguado JM, Mateu-de Antonio J, Gonzalez P, Del Castillo A (2007). Economic evaluation of linezolid versus teicoplanin for the treatment of infections caused by gram-positive microorganisms in Spain. J Chemother.

[30] Grau S, Mateu-de Antonio J, Soto J, Marin-Casino M, Salas E (2005). Pharmacoeconomic evaluation of linezolid versus teicoplanin in bacteremia by Gram-positive microorganisms. Pharm World Sci.

[31] Halpern MT, Palmer CS, Zodet M, Kirsch J (2002). Cost-effectiveness of gemifloxacin: results from the GLOBE study. Am J Health Syst Pharm.

[32] Jansen JP, Kumar R, Carmeli Y (2009). Cost-effectiveness evaluation of ertapenem versus piperacillin/tazobactam in the treatment of complicated intraabdominal infections accounting for antibiotic resistance. Value Health.

[33] Jansen JP, Kumar R, Carmeli Y (2009). Accounting for the development of antibacterial resistance in the cost effectiveness of ertapenem versus piperacillin/tazobactam in the treatment of diabetic foot infections in the UK. PharmacoEconomics.

[34] Kongnakorn T, Mwamburi M, Merchant S, Akhras K, Caro JJ, Nathwani D (2010). Economic evaluation of doripenem for the treatment of nosocomial pneumonia in the US: discrete event simulation. Curr Med Res Opin.

[35] Laohavaleeson S, Barriere SL, Nicolau DP, Kuti JL (2008). Cost-effectiveness of telavancin versus vancomycin for treatment of complicated skin and skin structure infections. Pharmacotherapy.

[36] Machado AR, Arns Cda C, Follador W, Guerra A (2005). Cost-effectiveness of linezolid versus vancomycin in mechanical ventilation-associated nosocomial pneumonia caused by methicillin-resistant *Staphylococcus aureus*. Braz J Infect Dis.

[37] Mullins CD, Kuznik A, Shaya FT, Obeidat NA, Levine AR, Liu LZ, Wong W (2006). Cost-effectiveness analysis of linezolid compared with vancomycin for the treatment of nosocomial pneumonia caused by methicillin-resistant *Staphylococcus aureus*. Clin Ther.

[38] Niederman MS, Chang JR, Stewart J, Asche CV, Lavin B, Nusrat R, Sullivan SD (2004). Hospitalization rates among patients with community-acquired pneumonia treated with telithromycin vs clarithromycin: results from two randomized, double-blind, clinical trials. Curr Med Res Opin.

[39] Niederman MS, Chang JR, Stewart J, Nusrat R, Nieman RB (2004). Comparison of hospitalization rates in patients with community-acquired pneumonia treated with 10 days of telithromycin or clarithromycin. Curr Med Res Opin.

[40] Patanwala AE, Erstad BL, Nix DE (2007). Cost-effectiveness of linezolid and vancomycin in the treatment of surgical site infections. Curr Med Res Opin.

[41] Sabes-Figuera R, Segu JL, Puig-Junoy J, Torres A (2008). Influence of bacterial resistances on the efficiency of antibiotic treatments for community-acquired pneumonia. Eur J Health Econ.

[42] Schurmann D, Sorensen SV, De Cock E, Duttagupta S, Resch A (2009). Cost-effectiveness of linezolid versus vancomycin for hospitalised patients with complicated skin and soft-tissue infections in Germany. Eur J Health Econ.

[43] Shorr AF, Susla GM, Kollef MH (2004). Linezolid for treatment of ventilator-associated pneumonia: a cost-effective alternative to vancomycin. Crit Care Med.

[44] Stein GE, Schooley SL, Havlichek DH, Nix DE (2008). Outpatient intravenous antibiotic therapy compared with oral linezolid in patients with skin and soft tissue infections: a pharmacoeconomic analysis. Infect Dis Clin Pract.

[45] Stranges PM, Hutton DW, Collins CD (2013). Cost-effectiveness analysis evaluating fidaxomicin versus oral vancomycin for the treatment of *Clostridium difficile* infection in the United States. Value Health.

[46] Tellier G, Chang JR, Asche CV, Lavin B, Stewart J, Sullivan SD (2004). Comparison of hospitalization rates in patients with community-acquired pneumonia treated with telithromycin for 5 or 7 days or clarithromycin for 10 days. Curr Med Res Opin.

[47] Vinken AG, Li JZ, Balan DA, Rittenhouse BE, Willke RJ, Goodman C (2003). Comparison of linezolid with oxacillin or vancomycin in the empiric treatment of cellulitis in US hospitals. Am J Ther.

[48] Zilberberg MD, Mody SH, Chen J, Shorr AF (2010). Cost-effectiveness model of empiric doripenem compared with imipenem-cilastatin in ventilator-associated pneumonia. Surg Infect (Larchmt).

[49] Grau S, Alvarez-Lerma F, del Castillo A, Neipp R, Rubio-Terres C (2005). Cost-effectiveness analysis of the treatment of ventilator-associated pneumonia with linezolid or vancomycin in Spain. J Chemother.

[50] Grau S, Lozano V, Valladares A, Cavanillas R, Xie Y, Nocea G (2014). Antibiotic expected effectiveness and cost under real life microbiology: evaluation of ertapenem and ceftriaxone in the treatment of community-acquired pneumonia for elderly patients in Spain. Clinicoecon Outcomes Res.

[51] Konijeti GG, Sauk J, Shrime MG, Gupta M, Ananthakrishnan AN (2014). Cost-effectiveness of competing strategies for management of recurrent *Clostridium difficile* infection: a decision analysis. Clin Infect Dis.

[52] McComb MN, Collins CD (2014). Comparative cost-effectiveness of alternative empiric antimicrobial treatment options for suspected enterococcal bacteremia. Pharmacotherapy.

[53] McGarry LJ, Merchant S, Nathwani D, Pawar V, DeLong K, Thompson D, Akhras K, Ingham M, Weinstein MC (2010). Economic assessment of doripenem versus imipenem in the treatment of ventilator-associated pneumonia. Journal of medical economics.

[54] Tan SC, Wang X, Wu B, Kang H, Li Q, Chen Y, Chen CI, Hajek P, Patel DA, Gao X (2014). Cost-effectiveness of linezolid versus vancomycin among patients with methicillin-resistant *staphylococcus aureus* confirmed nosocomial pneumonia in China. ViHRI.

[55] Vinken A, Li Z, Balan D, Rittenhouse B, Wilike R, Nathwani D (2001). Economic evaluation of linezolid, flucloxacillin and vancomycin in the empirical treatment of cellulitis in UK hospitals: a decision analytical model. J Hosp Infect.

[56] Wagner M, Lavoie L, Goetghebeur M (2014). Clinical and economic consequences of vancomycin and fidaxomicin for the treatment of *Clostridium difficile* infection in Canada. Can J Infect Dis Med Microbiol.

[57] Wilson SE, Turpin RS, Kumar RN, Itani KM, Jensen EH, Pellissier JM, Abramson MA (2008). Comparative costs of ertapenem and cefotetan as prophylaxis for elective colorectal surgery. Surg Infect (Larchmt).

[58] Bartsch SM, Umscheid CA, Fishman N, Lee BY (2013). Is fidaxomicin worth the cost? An economic analysis. Clin Infect Dis.

[59] Raftery J (2013). Value based pricing: can it work?. BMJ.

[60] Claxton K, Briggs A, Buxton MJ, Culyer AJ, McCabe C, Walker S, Sculpher MJ (2008). Value based pricing for NHS drugs: an opportunity not to be missed?. BMJ.

[61] Marsh K, Lanitis T, Neasham D, Orfanos P, Caro J (2014). Assessing the value of healthcare interventions using multi-criteria decision analysis: a review of the literature. PharmacoEconomics.

[62] Sussex J, Towse A, Devlin N (2013). Operationalizing value-based pricing of medicines: a taxonomy of approaches. PharmacoEconomics.

